# Effectiveness of mobile electronic devices in weight loss among overweight and obese populations: a systematic review and meta-analysis

**DOI:** 10.1186/s40608-014-0022-4

**Published:** 2014-10-14

**Authors:** Bushra Khokhar, Jessica Jones, Paul E Ronksley, Marni J Armstrong, Jeff Caird, Doreen Rabi

**Affiliations:** Institute of Public Health, University of Calgary, Calgary, Canada; W21C, University of Calgary, Calgary, Canada; Department of Clinical Epidemiology, Ottawa Hospital Research Institute (OHRI), Ottawa, Canada; Department of Cardiovascular and Respiratory Sciences, University of Calgary, Calgary, Canada

**Keywords:** Mobile electronic device, Weight loss, Obesity, Overweight

## Abstract

**Background:**

Mobile electronic devices, such as mobile phones and PDAs, have emerged as potentially useful tools in the facilitation and maintenance of weight loss. While RCTs have demonstrated a positive impact of mobile interventions, the extent to which mobile electronic devices are more effective than usual care methods is still being debated.

**Results:**

Electronic databases were systematically searched for RCTs evaluating the effectiveness of mobile electronic device interventions among overweight and obese adults. Weighted mean difference for change in body weight was the primary outcome. The search strategy yielded 559 citations and of the 108 potentially relevant studies, six met the criteria. A total of 632 participants were included in the six studies reporting a mean change in body weight. Using a random-effects model, the WMD for the effect of using mobile electronic devices on reduction in body weight was −1.09 kg (95% CI −2.12, −0.05). When stratified by the type of mobile electronic device used, it suggests that interventions using mobile phones were effective at achieving weight loss, WMD = −1.78 kg (95% CI −2.92, −0.63).

**Conclusions:**

This systematic review and meta-analysis suggests that mobile electronic devices have the potential to facilitate weight loss in overweight and obese populations, but further work is needed to understand if these interventions have sustained benefit and how we can make these mHealth tools most effective on a large scale. As the field of healthcare increasingly utilizes novel mobile technologies, the focus must not be on any one specific device but on the best possible use of these tools to measure and understand behavior. As mobile electronic devices continue to increase in popularity and the associated technology continues to advance, the potential for the use of mobile devices in global healthcare is enormous. More RCTs with larger sample sizes need to be conducted to look at the cost-effectiveness, technical and financial feasibility of adapting such mHealth interventions in a real clinical setting.

## Background

Finding effective strategies that promote healthy behavior change is an ongoing challenge [1]. The use of phone based interventions, mobile computing, and communication technology [mobile electronic devices] is rapidly expanding in healthcare and has demonstrated positive outcomes across a variety of populations [2,3]. Positive results include the creation of new paradigms for evaluation and deployment within the context of health care and health care management. “mHealth” is the overarching term used to describe the practice of medicine and public health, supported by mobile devices [4].

Use of mobile electronic devices has gained popularity in recent years as a tool to facilitate and maintain weight loss among overweight and obese populations. Worldwide, obesity has more than doubled since 1980 [5] and in several developed nations obesity accounts for 2% to 7% of total healthcare costs [6]. Although the majority of mHealth interventions are reported from high-income countries, there is emerging literature on the application of mobile technologies in low-income countries [3].

Mobile electronic devices have the potential to mimic the traditional in-person face-to-face healthcare provider consults providing a cost effective and convenient alternative. Based on the current primary literature there is potential for the use of mobile electronic interventions to facilitate weight loss in overweight and obese populations [4]. While Randomized Controlled Trials (RCTs) have demonstrated the potentially positive impact of mobile interventions, the extent to which mobile electronic devices are more effective than usual care methods is still being debated. The purpose of the present study was to perform a systematic review and meta-analysis of RCTs reporting the use of mobile electronic devices in weight loss efforts among overweight and obese adult population. Previous systematic reviews have been conducted on the topic of mHealth in the treatment of obesity and have highlighted the diverse populations and interventions that have been evaluated. To advance knowledge in this area, we conducted a meta-analysis and try to identify factors that might explain the disparate effect sizes described in these prior reviews [7-14].

This literature may still be young, but a systematic review and meta-analysis can help demonstrate early evidence of benefit or risk particularly when the literature is comprised of a small number of trials with limited statistical power.

## Methods

### Data sources and search strategy

This systematic review was performed using a predetermined, unpublished protocol and in accordance with standardized reporting guidelines [15]. Two reviewers (BK and JJ) performed independent searches of the following online electronic databases (Medline, PsycINFO, Embase and CENTRAL). The search of online databases was not restricted by language or date – the search is up to date to May 2014. The search was broken down into four main categories. To identify the relevant population, the first Boolean search was done using the term “OR” to explode [search by subject heading] and map [search by keyword] the following MeSH headings “overweight” or “obese”. To identify relevant interventions the second Boolean search used the term “OR” to explode and map “mobile phone” or “internet” or “computers handheld” or “wireless technology” or “text messaging” or “electronic mail” or “smartphone” or “[iPad or iPhone or iPod touch]” or “mHealth”. The third category of MeSH headings was also related to the intervention and included: “exercise” or “motor activity” or “physical fitness” or “diet”. Finally, the fourth group of key terms was used to identify study design. A Boolean search using the term “or” to explode and map the keywords “controlled clinical trials” or “randomized controlled trials” or “meta-analysis” or placebo*” or “random*” or “groups”. These four search categories were then combined using the Boolean operator “and”. In addition, two individuals (BK and JJ) searched the reference lists of prior review papers and all identified research articles were hand searched. Clinical trial registries were also consulted to identify all ongoing trials (www.clinicaltrials.gov, www.controlled-trials.com/mrct, www.isrctn.com). Table of contents of key journals [Telemedicine Journal and E-Health, Health Informatics Journal and Journal of Medical Internet Research] were also hand searched. Finally, experts identified during the review process were contacted for clarifications on their published trials.

### Study selection

Two reviewers (BK and JJ) independently evaluated articles for eligibility in a two-stage procedure. In stage one, all identified titles and abstracts were reviewed. In stage two, a full text review was performed on all of the articles that met the predefined inclusion criteria as well as all articles for which there was uncertainty as to eligibility. If either reviewer defined an article as eligible it was included in the full-text review and evaluated independently by both reviewers.

### Inclusion/exclusion criteria

An article was considered for inclusion in the systematic review if it met the following criteria: (i) study population (overweight or obese adults defined as having a BMI of ≥ 25.0 kg/m^2^); (ii) intervention (use of one or more mobile electronic devices); (iii) comparison (usual care defined as any weight loss intervention that does not use a mobile electronic device); (iv) outcome (change in BMI, weight and waist circumference) and (v) study design (RCT).

For inclusion in this review, trials had to report on original data (i.e. no review articles). Trials that used mobile electronic devices as a co-intervention to another weight loss intervention were included only if the data from the group using the mobile electronic device could be extracted independently and had a respective control. Mobile electronic devices considered for inclusion included, smartphones, Personal Digital Assistants (PDAs), portable media players, hand held video game consoles and handheld computers. Technologies that were not considered to be mobile electronic devices included: desktop personal computers, notebooks/sub-notebooks/netbooks, pagers, pedometers and landline telephones.

### Data extraction and quality assessment

Two reviewers (BK and JJ) independently extracted data from all trials that satisfied the inclusion criteria. Agreement between reviewers on the inclusion or exclusion of a document was assessed using Cohen’s kappa statistic [16]. Any disagreement in data extraction and/or study inclusion was resolved through discussion between the two reviewers and, when necessary, a third reviewer [PR]. Primary outcome was change in mean body weight. Baseline and post intervention means and standard deviations for mean body weight were extracted from both the intervention and control groups. The authors of potentially eligible trials were contacted when necessary to obtain missing or incomplete data.

A number of study characteristics were also extracted including geographic location, description of study population, primary outcomes, mobile electronic device used, description of intervention and control, results and participant feedback. Other data extracted included sample size, length of intervention in months, mean age, number and percentage of female participants, and features of the weight loss intervention.

Measures of study quality were also extracted including randomization, treatment allocation and concealment, blinding and loss to follow up. Study quality measures were scored independently by each reviewer (BK and JJ) and assessed using the validated 5-point scale described by Jadad [17].

#### Analysis

The mean difference was calculated by subtracting the mean body weight [in kg] at the end of follow up from the baseline body weight and was compared between groups. This allowed for a comparison of weight lost over and above what was lost in the control group, and not simply a comparison of weight lost in each study. The results were then weighted by sample size and the average taken [weighted mean difference (WMD)]. The WMD in each study was pooled using a random-effects model. Heterogeneity across RCTs was assessed visually by inspecting the I^2^ statistics. The I^2^ statistic quantifies the percentage of variability that can be attributed to between-study differences [18].

The results were stratified on study duration (less than or equal to six months and greater than six months) and by the type of mobile electronic device using the WMD for the effect of using mobile electronic devices on reduction in body weight. Finally, publication bias was assessed through visual inspection of funnel plots and Begg and Mazumdar’s [19] (rank correlation) test for asymmetry. All statistical analysis was performed using Stata, version 11.0 (Stata Corp., College Station, TX, USA).

## Results

### Study selection

**Figure 1:**
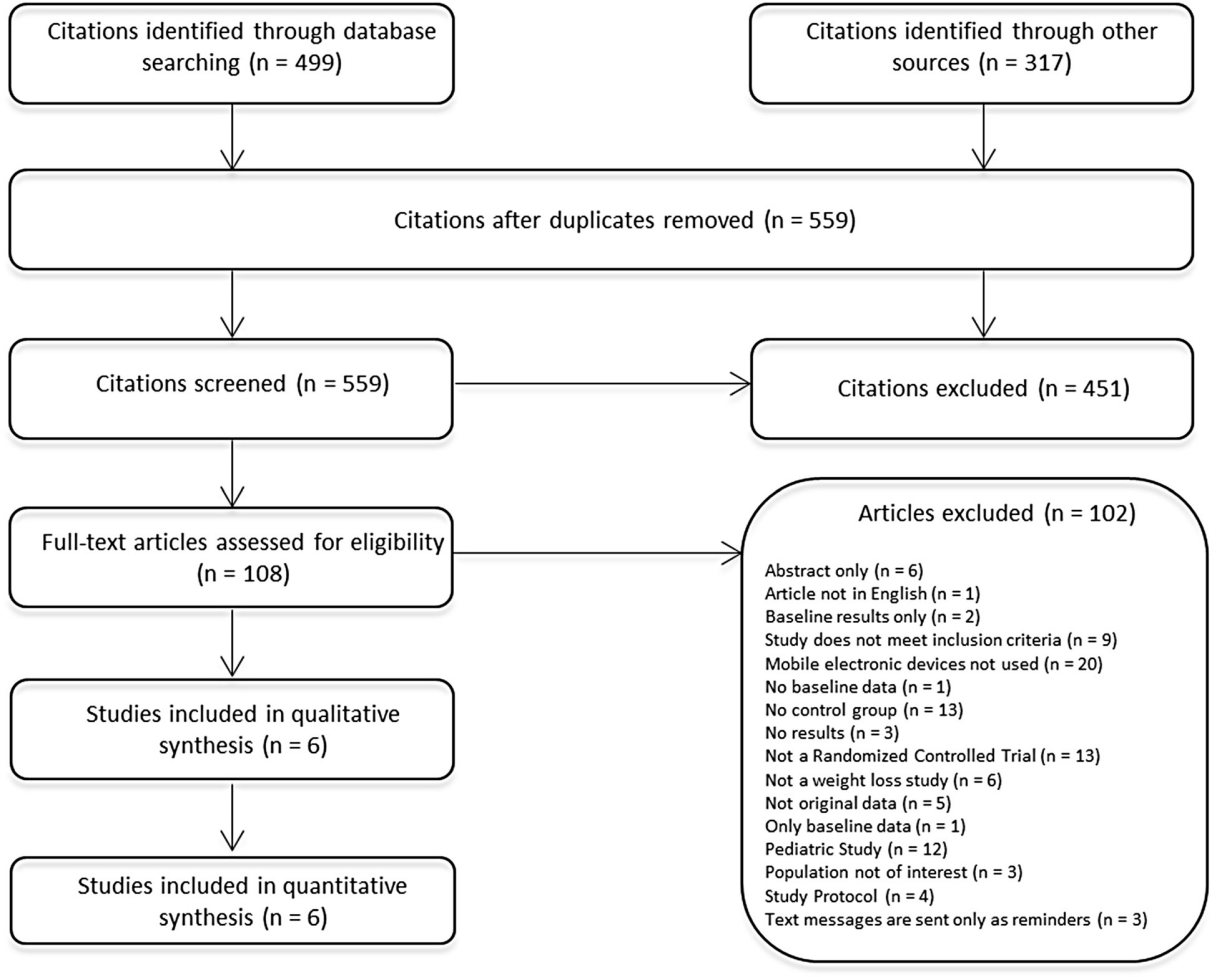
**Study flow chart.**

The initial search identified 559 unique citations. Through title and abstract review, we excluded 451 articles (k = 0.86). For the remaining 108 citations, full-text articles were obtained for more detailed evaluation. We excluded 102 articles during this screening phase primarily due to the lack of use of mobile electronic devices and not being an RCT. Six RCTs [k = 0.98] were deemed appropriate for inclusion for the review [20-25] and the purpose of meta-analysis (Figure 1).

### Study characteristics

The characteristics and weight outcomes of trials that met the inclusion criteria are shown in Table 1. A summary of the methodological details of the trials is presented in Table 2 and a summary of the study features including information on frequency of intervention usage, feedback, social support and adherence is shown in Table 3. Publication dates ranged from 2008 to 2013 with the number of participants per study varying from 65 to 174 and proportion of women from 65.43% to 84.80%. Mean baseline BMI ranged from 31 to 34.10 kg/m^2^ while mean age ranged from 38 to 53 years. Weight loss was the primary outcome in all trials; change in body weight (kilograms or pounds) was reported in all trials and two trials [20,21] reported change waist circumference. The mobile electronic devices used varied across trials, as did the study duration (ranged from 1 to 24 months). Three trials [21,22,24] used text messaging via mobile phones as the intervention and were compared to a control group who were given printed material, two trials [20,23] used PDAs and were compared to control groups who kept a paper diary and one trial [25] used a weight loss application on a smartphone and was compared to a control group who kept a paper diary.

**Table 1:** **Study characteristics**

**First author (Year)**	**Study duration (months)**	**Geographic location**	**Population**	**Primary outcome(s)**	**Mobile electronic device used**	**Description of intervention**	**Description of control**	**Results**	**User feedback**
Beasley (2008) [20]	1	United States	Overweight/obese adults. BMI = 25 to 40 kg/m^2^	Change in mean weight (lbs.), change in waist circumference (inches), adherence to intervention	Personal Digital Assistant (PDA)	PDA based program DietMatePro. Used as an alternative to paper based records	Paper diary	Mean weight decrease of 3.5 (SD = 4.9) lbs in the EG compared to 2.9 (SD = 4.8) lbs in the CG. Waist circumference decreased by 1.0 (SD = 1.2) inch in the EG compared to 0.5 (SD = 1.5) inch in the CG. Adherence was higher among the EG (43%) compared to the CG (28%)	N/A
Haapala (2009) [21]	12	Finland	Healthy overweight adults (18 to 59 years of age). BMI = 27 to 43 kg/m^2^	Change in body weight (kg) and waist circumference (cm)	Text messaging (Mobile Phone)	Use of mobile phone-operated weight-loss program, Weight Balance®. Program based on sending of various daily text messages	No intervention. Offered the studies weight-loss program free of charge after the 12-month study	Mean weight decrease of 4.5 (SD = 5.0) kg in the EG compared to 1.1 (SD = 5.8) kg in the CG. Waist circumference decrease by 6.3 (SD = 5.3) cm in the EG compared to 2.4 (SD = 5.4) cm in the CG	At 12 months, participants gave the program a score of 7.8 (0.8) on a user satisfaction scale from 4 to 10.
Patrick (2009) [22]	4	United States	Overweight adults (25 to 55 years of age). BMI = 25 to 39.9 kg/m^2^	Weight change (kg)	SMS & MMS messages (Mobile Phone)	Personalized SMS and MMS messages sent 2 to 5 times daily, printed materials, and brief monthly phone calls from a health counselor	Participants given monthly printed materials about weight control	Mean weight decrease of 2.46 (SD = 3.68) kg in the EG compared to 0.47 (SD = 3.62) kg in the CG.	At the end of the study, 22 of 24 (92%) of intervention participants said they would recommend the intervention for weight control to family and friends
Burke (2012) [23]	24	United States	Healthy overweight/obese adults (≤59 years of age). BMI = 27 to 43 kg/m^2^	Percent weight change (kg) at 24 months, adherence to self-monitoring over time	PDA	PDA with DietMatePro© software for self-monitoring.	Paper Diary and Nutritional reference book	Mean weight decrease of 1.18 (SD = 8.78) kg in the EG compared to 1.77 (SD = 7.23) kg in the CG. Weight loss was greater for those who were more adherent	N/A
Shapiro (2012) [25]	12	United States	Overweight adults (21 to 65 years of age). BMI ≥ 25 to 39.9 kg/m^2^	Weight change (lbs), adherence to intervention and steps per day	Text messaging (Mobile Phone)	SMS and MMS messages sent 2 to 5 times daily and monthly e-newsletters	Monthly e-newsletter	Mean weight decrease of 3.64 (SD = 12.01) lbs in the EG compared to 2.27 (SD = 9.39) lbs in the CG. Text-messaging adherence was moderately strong (60 to 69%). Participants’ steps increased almost 3000 steps/day over time (p < 0.05) in the EG.	Participants completed a survey assessing the program overall and its individual components. Likert rating scales were used: (e.g., ‘Please rate the SMS portion in enjoyability’ or ‘How likely would you be to refer the intervention to a friend?’ 0 = not at all, 5 = somewhat, 10 = extremely)
Carter (2013) [24]	6	United Kingdom	Overweight adults (18 to 65 years of age). BMI ≥ 27 kg/m^2^	Weight change (kg), change in body fat and adherence to intervention	Smartphone app (Mobile Phone)	MMM smartphone app for weight loss to be used on an Android operating system	Paper Diary	Mean weight decrease of 4.6 (SD = 5.20) kg in the EG compared to 2.9 (SD = 5.85) kg in the CG. Change in body fat was 1.3% (95% CI = −1.7 to −0.8) in the EG and 0.9% (95% CI = −1.5 to −0.4) in the CG	N/A

*BMI*: Body Mass Index. *lbs*: Pounds. *Kg*: Kilogram. *SMS*: Short Message Service. *MMS*: Multimedia Messaging Service. *PDA*: Personal Digital Assistant.

**Table 2:** **Characteristics of studies by outcome measure**

**First author (Year)**	**Sample size (n)**	**Study duration (months)**	***n*** **analyzed (loss to follow-up,%)**	**Mean age (Years)**	***n*** **female (%)**	**Outcome**	**Experimental group, mean difference, kg (SD)**	**Control group, mean difference, kg (SD)**
Beasley (2008) [20]	174	1	159 (8.62%)	53	140 (80.07%)	Weight (kg)	1.59 (2.23)	1.32 (2.18)
Haapala (2009) [21]	124	12	82 (33.87)	38.05	96 (77.42%)	Weight (kg)	4.50 (5.00)	1.10 (5.80)
Patrick (2009) [22]	65	4	52 (20.00%)	44.9	52 (80.00%)	Weight (kg)	2.46 (3.68)	0.47 (3.62)
Burke (2012) [23]	140	24	121 (13.57%)	46.8	119 (84.80%)	Weight (kg)	1.18 (8.78)	1.77 (7.23)
Shapiro (2012) [25]	170	12	130 (23.53%)	42	111 (65.43%)	Weight (kg)	1.66 (5.46)	1.03 (4.27)
Carter (2013) [24]	86	6	60 (30.23%)	41.85	66 (76.74%)	Weight (kg)	4.60 (5.20)	2.90 (5.85)

**Table 3:** **Study features**

	**Study duration ≤ 6 months**			**Study duration > 6 months**		
	**Beasley**	**Patrick**	**Carter**	**Shapiro**	**Haapala**	**Burke**
**Reported effect size, WMD (95% CI)**	**−0.27 (−0.93, 0.39)**	**−1.99 (−3.76, −0.22)**	**−1.70 (−4.73, 1.33)**	**−0.63 (−2.35, 1.09)**	**−3.40 (−5.75, −1.05)**	**0.59 (−2.28, 3.46)**
**Study feature**						
Self-monitoring (Weight)	✓	✓	✓	✓	✓	✓
Participants asked to report weight frequently	✓	✓	x	✓	✓	x
Counselor/Human Professional feedback and communication	x	✓	x	x	x	x
Automatic feedback given	x	✓	✓	✓	✓	x
Prompted reminder to record meals or weigh themselves	✓	x	✓	✓	x	x
Social support	x	x	✓	x	x	x
Opportunity for peer support	x	x	✓	x	x	x
Structured program	✓	✓	x	x	✓	✓
Individually tailored	✓	✓	x	✓	✓	✓
Goal includes to reduce calorie intake	✓	✓	✓	✓	✓	✓
Adherence to dietary monitoring measured	✓	✓	✓	✓	x	✓
Tailored diet/exercise prescription	Each participant was provided with an individual target calorie level.	A baseline dietary assessment for each participant was used to identify unique diet behavior challenges that may contribute to increased caloric intake (eg, snacking behaviors, pacing of consumption, and self-monitoring of food intake). The server processed these data to create goals to target based upon particular logic rules of the expert system. These goals were then presented to the user via text or MMS messages to serve as prompts for food selection and behavioral improvements.	-	-	The program calculated the dieter’s daily energy requirements and physical activity.	-
Additional support	Food portion education pamphlet to assist in determining appropriate food portion sizes for recording using either assessment method.	At intervention onset, participants were given a printed binder with nutrition topics and behavioral strategies to supplement the phone-based messaging and a food and exercise journal to support self-monitoring.	-	Participants received monthly e-newsletters with diet and physical activity information from credible publicly available sources. They also had access to a website that provided health tips, recipes, food and physical activity logs, and a personal weight chart. Participants received USDA recommendations for a balanced diet.	-	Participants daily energy consumption goals were 1200 to 1800 calories, based on the weight and gender; ≤ 25%of total calories could ne form fat. Weekly physical activity goal was 180 minutes by 6 months and increase by 30 minutes semi-annually.

### Study features

Table 3 describes features of the intervention used in each of the six RCTs. All the trials were self-monitored interventions with the goal of reducing calorie intake to promote weight loss. Participants were required to report their weight frequently in four trials [20-22,24]. Counselor [healthcare professional] feedback was provided in only one trial [22], whereas automated feedback was provided in four trials [21,22,24,25]. Three trials [20,24,25] used reminder prompts to encourage participants to record meals or weigh themselves. Only one trial [25] provided social and peer support. Four trials [20-23] followed a structured program. Almost all of them were individually tailored, expect for one [25], and measured for intervention adherence, except for one [21].

### Quality assessment

The quality of trials according to the Jadad score [17] was low to moderate as shown in Table 4. Three trials [21,22,24] reported allocation concealment and blinding was reported in two trials [24]. However, all trials reported on intention-to-treat analysis. Common sources of potential bias included research staff not blinded to the treatment groups, and unclear description of randomization. All trials adequately described dropouts, except one [22].

**Table 4:** **Study quality characteristics**

**Study (first author, year)**	**Inclusion/exclusion criteria**	**Randomization described**	**Allocation concealment**	**Blinding**	**Intent to treat analysis**	**Loss to follow up described**	**Loss to follow up (%)**	**Jaded scoring**
Beasley (2008) [20]	Yes	Unclear	No	Unclear	Yes	Yes	Not reported	2
Haapala (2009) [21]	Yes	Unclear	Yes	Unclear	Yes	Yes	27% - EG	3
							35% - control	
Patrick (2009) [22]	Yes	Unclear	Yes	No	Yes	Unclear	Not reported	2
Burke (2012) [23]	Yes	Yes	Unclear	Unclear	Yes	Yes	13.9% - EG	3
							13.2% - CG	
Shapiro (2012) [25]	Yes	Yes	Yes	Yes	Yes	Yes	30% - EG	3
							18% - CG	
Carter (2013) [24]	Yes	Unclear	Unclear	Yes	Yes	Yes	7% - EG	2
							47% - CG	

EG: Experimental Group.

CG: Control Group.

### Risk of publication bias

The risk of publication bias of the included trials was assessed through visual inspection of funnel plots and Begg and Mazumdar’s [19] [rank correlation] test for asymmetry. There was no evidence of publication bias with Begg and Mazumdar’s test or with visual inspection of the funnel plots.

### Effect of use of mobile electronic devices on body weight

A total of 632 participants were included in the six RCTs reporting a mean change in body weight. There were 320 participants who used some form of a mobile electronic device and 312 control comparator participants. All trials reported change in body weight and were pooled to assess effect estimates. All the trials demonstrated a weight loss as a result of using a mobile electronic device-assisted intervention (Table 2), but the effect sizes and the contents of the individual interventions varied greatly.

**Figure 2:**
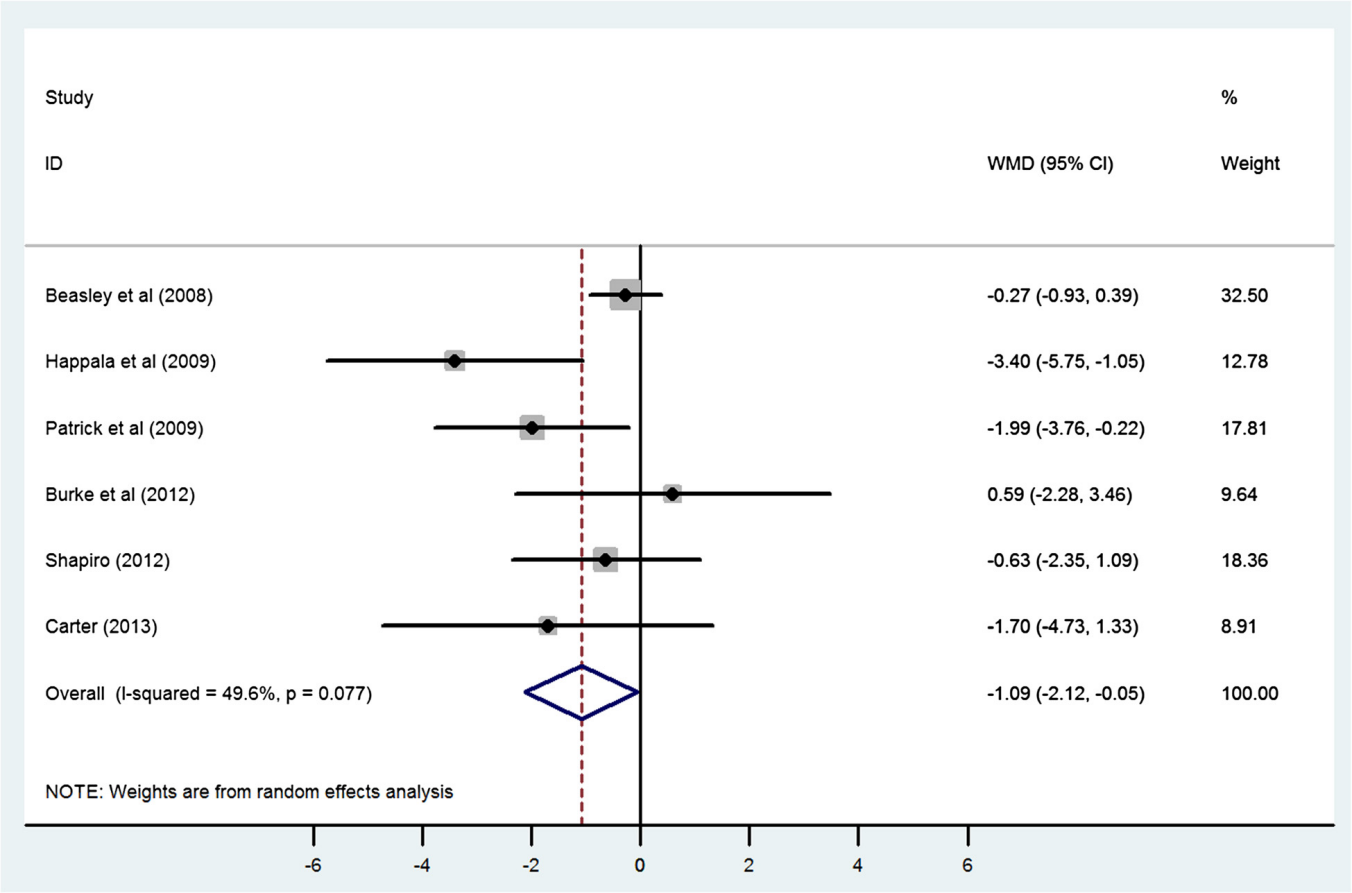
**Meta-analysis of standardized change scores in body mass in mobile electronic device users group compared with control.** Degree of shading corresponds with study weighting in random-effects model. WMD, weighted mean difference.

**Figure 3:**
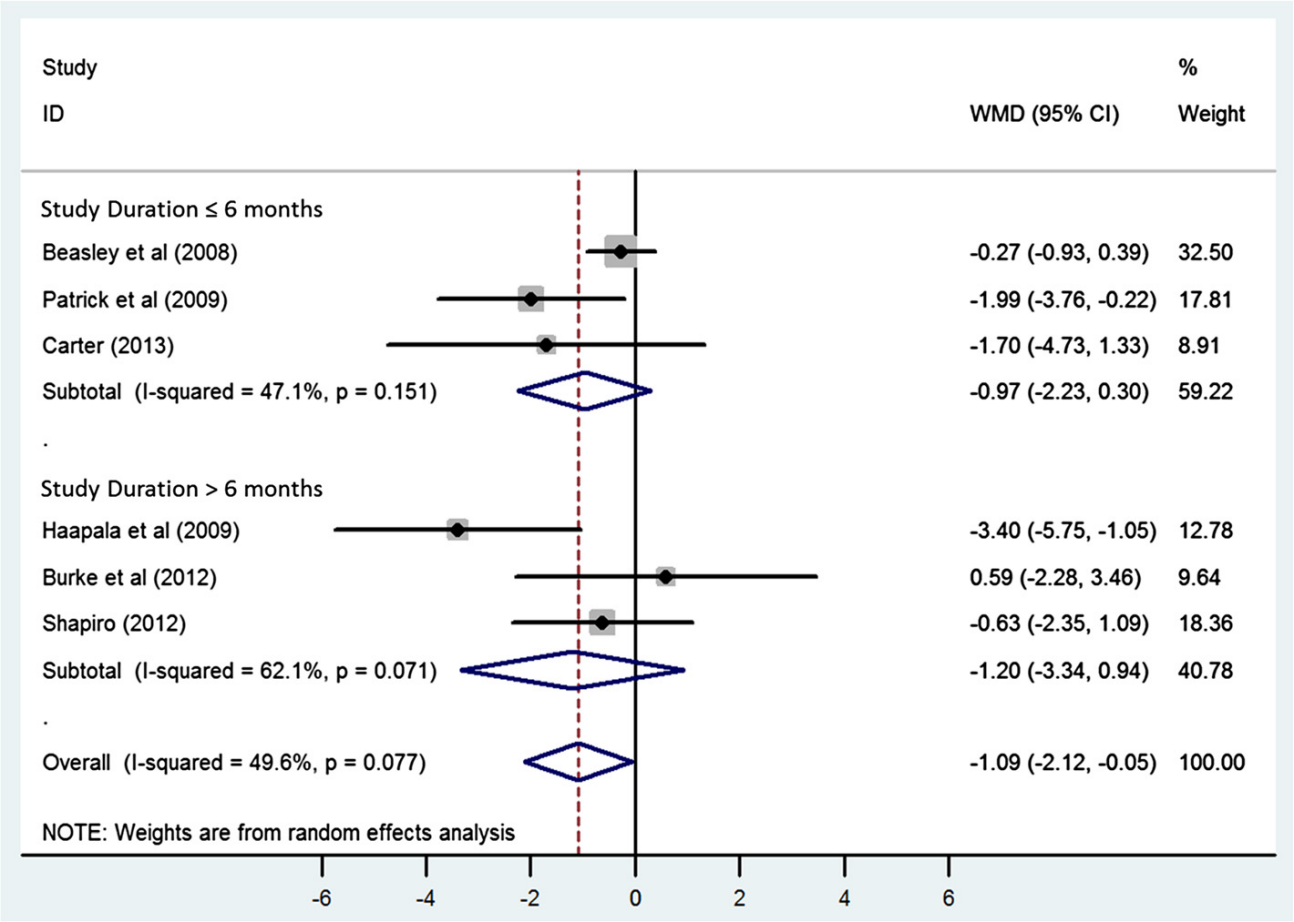
**Meta-analysis of standardized change scores in body mass in mobile electronic device users group compared with control, stratified by the study duration.** Degree of shading corresponds with study weighting in random-effects model. WMD, weighted mean difference.

**Figure 4:**
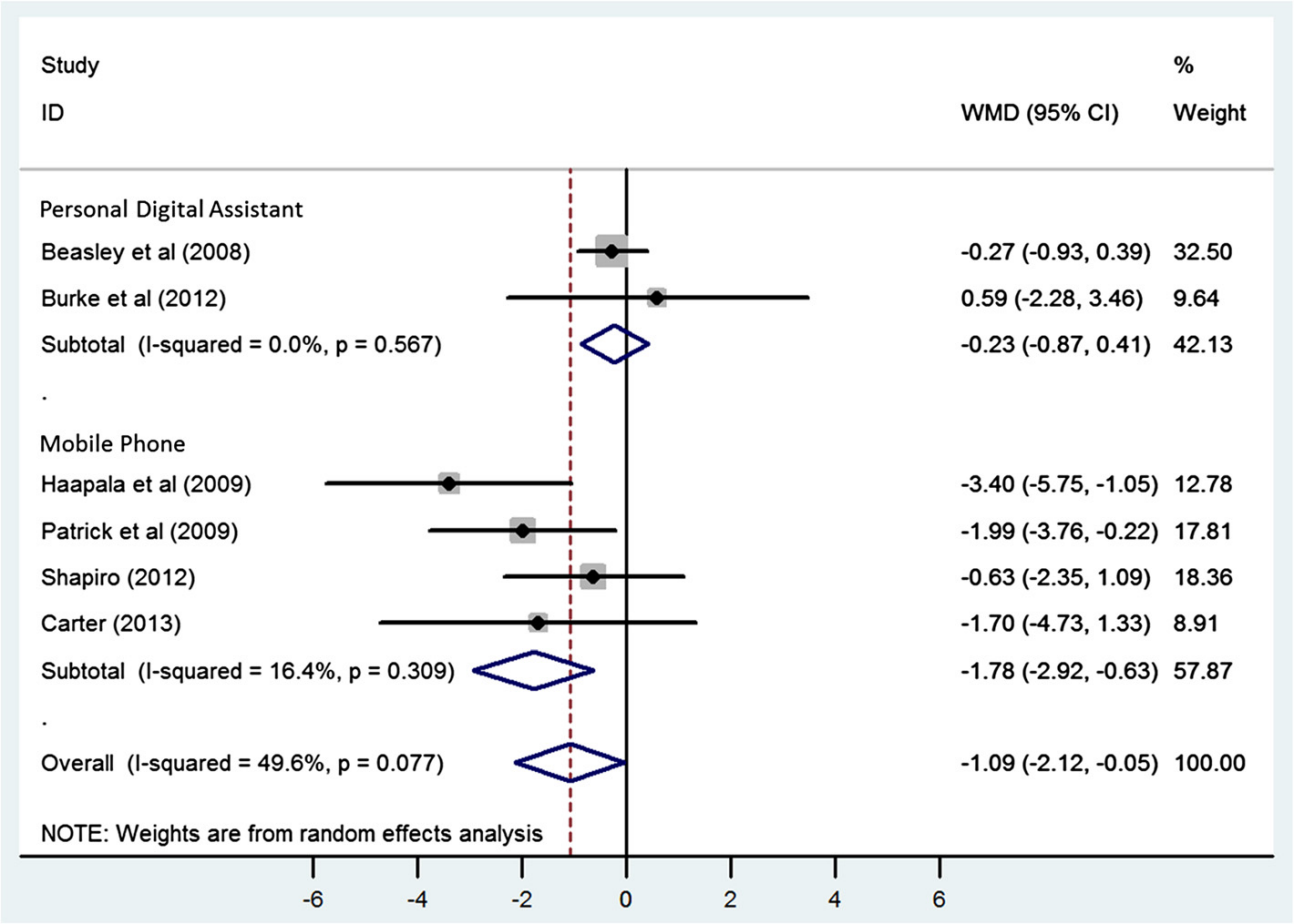
**Meta-analysis of standardized change scores in body mass in mobile electronic device users group compared with control, stratified by the type of mobile electronic device used.** Degree of shading corresponds with study weighting in random-effects model. WMD, weighted mean difference.

Using a random-effects model, the WMD for the effect of using mobile electronic devices on reduction in body weight was −1.09 kg (95% CI −2.12, −0.05) in the six eligible trials (Figure 2). Heterogeneity was observed in this pooled estimate (I^2^ = 49.60%, p value = 0.077). Stratified analyses were conducted to determine whether duration of the intervention or the device used to deliver the intervention (PDA vs. mobile phone) were associated with differing effect sizes or measures of heterogeneity. When stratified on study duration, there was no appreciable difference between studies that were 6 months or less in duration (WMD = −0.97, 95% CI −2.23, 0.30) or those that were longer than 6 months (WMD = −1.20 kg, 95% CI −3.34, 0.94), with both strata demonstrating non-significant reductions in weight with a moderate degree of heterogeneity (I^2^ = 47.1%, p value = 0.151 for trials ≤ six months and I^2^ = 62.1%, p value = 0.071 for studies > six months) (Figure 3). However, when stratified by the type of mobile electronic device used, PDA or mobile phones, the heterogeneity reduced in the group that used an intervention delivered via mobile phones (I^2^ = 16.40%, p value = 0.309) and studies that used mobile phones were found to have a significant and consistent benefit with respect to weight loss (WMD = −1.78, 95% CI −2.92, −0.63) (Figure 4). One of the trials [25] in the strata using mobile phones was using a weight loss application on a smart phone whereas the remaining three trials used text messaging via mobile phones. Therefore a sensitivity analysis was performed to look at the robustness of the pooled estimate. When the trial using a smart phone application was excluded, the WMD for the effect of using mobile electronic devices on reduction in body weight was −1.05 kg (95% CI −2.20, 0.10), suggesting that the benefit associated with the single study using the mobile application strongly influenced the pooled effect size.

## Discussion

This systematic review summarizes the results for the effectiveness of mobile electronic devices in weight loss among overweight and obese populations. Our meta-analysis of these data suggests that interventions using mobile phones were effective at achieving weight loss. Moreover, the data was very consistent, with low statistical heterogeneity suggesting that to date, trials evaluating mobile phone interventions for weight loss show remarkably consistent weight loss benefit even though the content of the interventions varied from study to study. Obesity is a condition that requires lifelong management and monitoring, and interventions with sustained benefits are sorely needed. Whether mHealth interventions result in sustained weight loss is not known, but given the very high prevalence of overweight and obesity and the relative low cost and high accessibility of mHealth tools, the results of this meta-analysis are encouraging and support further investigation and evaluation of the long-term efficacy of mHealth tools in weight loss.

Our results align with those of others that have evaluated healthy behavior change in other populations. The use of mobile phone messaging intervention has been shown to be an effective technique for lifestyle modification to reduce incidence of type 2 diabetes in men, with a BMI of 23 kg/m^2^ or higher, in India [26]. Even though there was no significant effect of the intervention on BMI, the cumulative incidence of type 2 diabetes was lower in those who received the mobile phone messaging intervention [26]. In addition to diabetes management [27], the use of mobile electronic devices been shown to be an effective means to promote healthy behaviors including smoking cessation [28], preventive medication adherence [29] and asthma management [30]. In this meta-analysis, there are three forms of mHealth interventions being used, mobile phone text messaging, mobile phone applications and PDAs. The feasibility of using text messaging to effect behavior changes that promote healthy behavior has been demonstrated [31] however, more information is needed on what combinations of text message factors (dose, duration, complimentary technologies, etc.) produce the best results. Similarly, there is evidence to support the notion that the use of PDAs outperform paper methods for data collection and led to improved compliance to study protocol and was preferred by the participants over paper [32]. However, an increased accuracy of data entry cannot be assumed, but it is possible to make data entry more accurate by carefully structuring weight loss questions to allow only determinate types of responses and by using prompts to ensure that questions are followed in sequence and are not skipped and date stamped [33]. Our analysis provides little evidence that the use of PDAs in and of themselves is helpful in promoting weight loss.

As the use of mobile technology in physical activity research among overweight and obese population is still fairly new, this review is foundational in informing the development of appropriate and efficient mobile intervention techniques to enhance weight loss in those overweight and obese. Currently, the literature surrounding mHealth and diabetes management [24] seems to be most advanced while the evidence base for other health topics is sparse. The most unique and advantageous feature of mobile electronic devices is their increasing popularity and ease of use. In particular, smart phones and their web-applications [“apps”] provide constant connectivity to store data locally or exchange it via the Internet. Since 2008, application development has continued to grow across multiple platforms at an exponential rate and has mirrored the increase in smart phone users worldwide [34]. While our review identified only a single study that evaluated a mobile phone weight loss application [25], this study did have positive results and should encourage future work in this area.

In addition to improving healthcare outcomes, the use of mobile electronic devices may help reduce disparities [2]. In our current review the majority of included studies originate from the US, however previous systematic reviews have discussed and demonstrated the international applicability of mobile phone technology [2]. Individuals around the world are using mobile technologies to access health services and information and health professionals are formally and informally integrating mobile technologies into public health and clinical activities [35]. Today, the ownership and use of mobile phones has become just as prevalent among persons of low socioeconomic status as those from the general population and therefore, the use of mobile technology has the potential to reduce income-related disparities [2,3].

### Strengths and limitations

A number of limitations of the present review should be acknowledged. In terms of study quality, half of the included studies lacked allocation concealment and/or blinding, which may introduce bias in the estimation of the effect of the use of mobile electronic devices. The number of participants was small, ranging from 75 to 174 in the six RCTs included and this may have limited our statistical power. The population of interest for this study was ‘healthy participants’ ruling out the opportunity to address those populations with chronic diseases associated with obesity such diabetes and cardiovascular conditions. The population of interest may also belong to a certain literacy and socioeconomic status because of the use of mobile phone and Internet in the experimental groups. Our overall pooled estimates of effect were associated with a large degree of heterogeneity. However, our stratified analyses were successful in identifying type of mobile electronic device and the duration of intervention as potential sources of heterogeneity in this review.

Despite these limitations, this systematic review and meta-analysis includes the most recently published studies using mobile electronic devices using PRISMA guidelines [15]. While our results suggest a weight loss benefit with mHealth interventions, the specific elements of these interventions that affect healthy behavior change are not fully understood. To advance knowledge and enhance understanding of the benefits of using mobile electronic devices, standardization of evaluative studies in this area is important. Results from this systematic review and meta-analysis should be considered in future trial designs.

### Recommendations for improving the quality and reporting of future trials

This review demonstrates the need for more systematic study design and standardized reporting to enable comparisons across different mHealth interventions. Associations between study features such as providing counseling by a healthcare professional, automated feedback, measuring adherence to use of intervention, prompting reminder to log food, providing financial incentives to participants, tailored diet and exercise prescriptions and weight loss should be studied further. Understanding these associations will enable efficient distribution of resources during trials. For example, it would be financially beneficial to understand if an expensive resource such as counseling by a healthcare professional is necessary for the trial or an automated feedback in the context of a tailored intervention, is equally beneficial. Tailored interventions have previously shown to be an appropriate model for weight management according to principles suggested by Social Cognitive Theory and the Social Marketing Model [36].

## Conclusion

This systematic review and meta-analysis suggests that mobile electronic devices have the potential to facilitate weight loss in overweight and obese populations, but further work is needed to understand if these interventions have sustained benefit and how we can make these mHealth tools most effective on a large scale. As the field of healthcare increasingly utilizes novel mobile technologies, the focus must not be on any one specific device but on the best possible use of these tools to measure and understand behavior. As mobile electronic devices continue to increase in popularity and the associated technology continues to advance, the potential for the use of mobile devices in global healthcare is enormous. More controlled studies with larger sample sizes need to be conducted to look at the cost-effectiveness, technical and financial feasibility of adapting such mHealth interventions in a real clinical setting.

## References

[1] Care Continuum Alliance. *Care Continuum Alliance (CCA) definition of disease management*. Retrieved 2012-09-30

[2] Krishna S, Boren SA, Balas EA (2009). Healthcare via cell phones: a systematic review. Telemedicine and e-Health.

[3] Koivusilta LK, Lintonen TP, Rimpela AH (2007). Orientation in adolescent use of information and communication technology: a digital divide by sociodemographic background, educational career, and health. Scand J Public Health.

[4] Free C, Phillips G, Felix L, Galli L, Patel V, Edwards P (2010). The effectiveness of M-health technologies for improving health and health services: a systematic review protocol. BMC Res Notes.

[5] World Health Organization: *10 Facts on physical activity*; http://www.who.int/features/factfiles/physical_activity/en. Accessed September 26, 2012.

[6] Withrow D, Alter DA (2011). The economic burden of obesity worldwide: a systematic review of the direct costs of obesity. Obes Rev.

[7] Wieland LS, Falzon L, Sciamanna CN, Trudeau KJ, Brodney Folse S, Schwartz JE, Davidson KW (2012). Interactive computer-based interventions for weight loss or weight maintenance in overweight or obese people. Cochrane Database Sys Rev.

[8] Neve M, Morgan PJ, Jones PR, Collins CE (2010). Effectiveness of web-based interventions in achieving weight loss and weight loss maintenance in overweight and obese adults: a systematic review with meta-analysis. Obes Rev.

[9] Fanning J, Mullen SP, McAuley E (2012). Increasing physical activity with mobile devices: a meta-analysis. J Med Int Res.

[10] Bacigalupo R, Cudd P, Littlewood C, Bissell P, Hawley MS, Buckley WH (2013). Interventions employing mobile technology for overweight and obesity: an early systematic review of randomized controlled trials. Obes Rev.

[11] Bort-Roig J, Gilson ND, Puig-Ribera A, Contreras RS, Trost SG (2014). Measuring and influencing physical activity with smartphone technology: a systematic review. Sports Med.

[12] Siopis G, Chey T. & Allman-Farinelli M. (2014) **A systematic review and meta-analysis of interventions for weight management using text messaging.** *J Hum Nutr Diet*. doi:10.1111/jhn.12207 10.1111/jhn.12207 24480032

[13] Stephens J, Allen J (2013). Mobile Phone interventions to increase physical activity and reduce weight: a systematic review. J Cardiovasc Nurs.

[14] Coons MJ, Demott A, Buscemi J, Duncan JM, Pellegrini CA, Steglitz J, Pictor A, Spring B (2012). Technology interventions to curb obesity: a systematic review of the current literature. Curr Cardiovasc Risk Reports.

[15] Liberati A, Altman DG, Tezlaff J, Mulrow C, Gotzsche PC, Ioannidis JPA, Clarke M, Devereaux PJ, Kleijnen J, Moher D (2009). The PRISMA statement for reporting systematic reviews and meta-analyses of studies that evaluate health care interventions: explanation and elaboration. Annals Int Med.

[16] Cohen J (1960). A coefficient of agreement for nominal scales. Educ Psychol Meas.

[17] Jadad AR, Moore AR, Carroll D, Jenkinson C, Reynolds DJ, Gavaghan DJ, McQuay HJ (1996). Assessing the quality of reports of randomized clinical trials: is blinding necessary?. Control Clin Trials.

[18] Higgins JPT, Thompson SG (2002). Quantifying heterogeneity in a meta-analysis. Stat Med.

[19] Begg CB, Mazumdar M (1994). Operating characteristics of a rank correlation test for publication bias. Biometrics.

[20] Beasley JM, Riley WT, Davis A, Singh J (2008). Evaluation of a PDA-based dietary assessment and intervention program: a randomized controlled trial. Am J Nutr.

[21] Haapala I, Barengo NC, Biggs S, Surakka L, Manninen P (2009). Weight loss by mobile phone: a 1-year effectiveness study. Pub Health Nut.

[22] Patrick K, Raab F, Adams MA, Dillon L, Zabinski M, Rock CL, Griswold WG, Norman GJ (2009). A text message-based intervention for weight loss: randomized controlled trial. J Med Internet Res.

[23] Burke LE, Styn MA, Sereika SM, Conroy MB, Ye L, Glanz K, Sevick MA, Ewing LJ (2012). Using mHealth technology to enhance self-monitoring for weight loss: a randomized trial. Am J Prev Med.

[24] Carter MC, Burley VJ, Nykjaer C, Cade JE (2013). Adherence to a smartphone application for weight loss compared to website and paper diary: Pilot randomized controlled trial. J Med Internet Res.

[25] Shapiro JR, Koro T, Doran N, Thompson S, Sallis JF, Calfas K, Patrick K (2012). Text4Diet: a randomized controlled study using text messaging for weight loss behaviors. Prev Med.

[26] Ramachandran A, Snehalatha C, Ram J, Selvam S, Simon M, Nanditha A, Shetty AS, Godsland IF, Chaturvedi N, Majeed A, Oliver N, Toumazou C, Alberti KG, Johnston DG (2013). Effectiveness of mobile phone messaging in prevention of type 2 diabetes by lifestyle modification in men in India: a prospective, parallel-group, randomized controlled trial. Lancet Diab Endocrinol.

[27] Liang X, Wang Q, Yang X, Cao J, Chen J, Mo X, Huang J, Wang L, Gu D (2011). Effect of mobile phone intervention for diabetes on glycemic control: a meta-analysis. Diabet Med.

[28] Whittaker R, Borland R, Bullen C, Lin R, McRobbie H, Rodgers A (2009). Mobile phone-based interventions for smoking cessation. Cochrane Database Syst Rev.

[29] Cocosila M, Archer N, Haynes RB, Yuan Y (2009). Can wireless text messaging improve adherence to preventive activities? results of a randomized controlled trial. Int J Med Inform.

[30] Ostojic V, Cvoriscec B, Ostojic SB, Reznikoff D, Stipic-Markovic A, Tudjman Z (2005). Improving asthma control through telemedicine: a study of short-message service. Telemed J E Health.

[31] Cole-Lewis H, Kershaw T (2010). Text messaging as a tool for behavior change in disease prevention and management. Epi Rev.

[32] Oystein D, Hagen KB (2007). Despite technical problems personal digital assistants outperform pen and paper when collecting patient diary data. J Clin Epi.

[33] Lane SJ, Heddle NM, Arnold E, Walker I (2006). A review of randomized controlled trials comparing the effectiveness of hand held computers with paper methods for data collection. BMC Med Inform Decis Mak.

[34] Purcell, Kristen. **Half of Adult Cell Phone Owners Have Apps on Their Phones.** Rep. Washington: Pew Internet, 2011.

[35] Mechael PN (2009). The case for mHealth in developing Countries. Innov Technol Governance Globalization.

[36] Tufano JT, Karras BT (2005). Mobile eHealth interventions for obesity: a timely opportunity to leverage convergence trends. J Med Internet Res.

